# Genome Sequence of *Alcaligenes* sp. Strain HPC1271

**DOI:** 10.1128/genomeA.00235-12

**Published:** 2013-02-21

**Authors:** Atya Kapley, Sneha Sagarkar, Himgouri Tanksale, Nandita Sharma, Asifa Qureshi, Anshuman Khardenavis, Hemant J. Purohit

**Affiliations:** Environmental Genomics Division, National Environmental Engineering Research Institute, Nehru Marg, Nagpur, India

## Abstract

We report a draft genome sequence of *Alcaligenes* sp. strain HPC1271, which demonstrates antimicrobial activity against multidrug-resistant bacteria. Antibiotic production by *Alcaligenes * has not been frequently reported, and hence, the availability of the genome sequence should enable us to explore new antibiotic-producing gene clusters.

## GENOME ANNOUNCEMENT

*Alcaligenes* sp. strain HPC1271 was isolated from activated sludge of a common effluent treatment plant that treats industrial wastewater. This bacterial isolate demonstrates antimicrobial activity against two multidrug-resistant strains: *Enterobacter* sp., resistant to sulfamethoxazole, ampicillin, azithromycin, and tetracycline, and *Serratia* sp. GMX, resistant to sulfamethoxazole, ampicillin, azithromycin, tetracycline, and netilmycin.

*Alcaligenes* sp. HPC1271 was grown in LB broth at 30°C at 150 rpm. Cells in the late-log phase of growth were harvested and total DNA was prepared as reported earlier (1). DNA purity was measured by the *A*_260_ to *A*_280_ ratio with the NanoDrop 1000 Spectrophotometer (Thermo Scientific). The genome was sequenced using the Ion Torrent sequencing platform (Applied Biosystems). A total of 1,303,717 reads were generated, of which 1,233,999 reads were able to be assembled using MIRA (v3.4) into 78 contigs. Genome coverage was 51.94-fold. The genome was annotated using the NCBI Prokaryotic Genomes Automatic Annotation Pipeline (PGAAP) and was independently analyzed on the Rapid Annotations using Subsystems Technology (RAST) server (2). The information contained in the draft genome is comprised of 4,270,933 bp sequence data, with a mean G+C content of 56.6%. Seventy-eight contigs cover a total of 3,914 genes, 57 tRNAs, 7 rRNAs, and 3,834 proteins.

The sequence of the 16S rRNA gene (1,504 bp) of *Alcaligenes* sp. HPC1271 is 99% identical to those of *A. faecalis* strain IAM12369 (ATCC 8750; NCBI accession no. NR_043445) and *A. faecalis* subsp. *faecalis* strain NCIB 8687 (NCBI accession no. AKMR00000000). A 16S RNA gene is present within a “ribosomal operon” with the genes for 23S rRNA, tRNA-Ala(TGC), and tRNA-Ile(GAT). In total, 125 tandem repeats are present in the genome, with a maximum of 10 repeats in contig 3. The largest contig is the third one, which is 291,167 bp.

While exploring the preliminary annotation data of the *Alcaligenes* sp. HPC1271 genome for genes related to possible antibiotic production, we found a type I nonribosomal polyketide synthetase (enterobactin synthase) gene in contig 51 (bp 155388 to 158867), a type I polyketide synthetase (PKS) cluster in contig 54 (bp 79481 to 80971), and a colicin synthase cluster in contig 14 (bp 16262 to 26640). We postulate that these genes may be responsible for antibiotic production, leading to the antimicrobial activity we observed. However, further studies are required to confirm this. Several unknown gene clusters were also found in the HPC1271 genome.

Bacteria from the genus *Alcaligenes* are reported in the commercial production of some useful secondary compounds, like nonstandard amino acids and the biopolymer polyhydroxybutyrate. Very few reports are available on the production of antibiotics from the members of this genus (3). There are two draft genomes of *Alcaligenes* sp. available in GenBank (4). With this strain demonstrating antimicrobial activity against multidrug-resistant bacteria, it is of interest to analyze these sequence data for new antibiotic-producing genes.

### Nucleotide sequence accession numbers. 

The draft genome of *Alcaligenes* sp. HPC1271 was deposited in GenBank under accession no. AMXV00000000 (AMXV01000001 to AMXV01000078).
